# The Values of the Care Economy: Comment on "Ensuring Global Health Equity in a Post-pandemic Economy"

**DOI:** 10.34172/ijhpm.2023.7762

**Published:** 2023-08-20

**Authors:** Jennifer Cohen

**Affiliations:** ^1^Department of Global and Intercultural Studies, Miami University, Oxford, OH, USA; ^2^Ezintsha, Faculty of Health Sciences, University of the Witwatersrand, Johannesburg, South Africa

**Keywords:** Care Work, Inequality, Inequity, Capitalism, Gender, COVID-19

## Abstract

Labonté proposes that health equity and environmental sustainability may be best obtained through a care economy. Because a care economy plays a key role in Labonté’s formulation, its position in the capitalist political economy, the work it entails, and the workers who do it all merit further reflection. I aim to complement Labonté’s editorial by elaborating on care economies and the work of social reproduction. The existing care economy is a structural part of capitalism that largely generates and sustains inequities, reinforcing Labonté’s argument that transformation is needed. Transformation could, and should, change the perceived value, status, and material rewards of work in the care economy. I then touch on the policy tools Labonté describes, highlighting how they connect to my broader point: that the care economy is currently an integral, but devalued part of capitalism. For a transformation to take place, raising perceived value, status, and material rewards of caring work and the people who do it must be an explicit policy goal.

## Introduction

 In his insightful editorial, Ronald Labonté writes that the COVID-19 pandemic demonstrated that socioeconomic inequality is lethal.^1^ I agree. Recognizing that the pandemic is internal to capitalism sharpens the contradictions between a world shaped by the profit motive and health justice.^2^ Labonté identifies capitalism’s economic growth imperative as the underlying problem. As such, reforms like stakeholder capitalism, the pursuit of capitalist ‘green recoveries,’ and policy options that could improve well-being within the growth paradigm cannot address the problem. He concludes that a transformative shift away from an economic system centered on economic growth to one that enhances health, prosperity, and well-being is necessary. Labonté proposes that health equity and environmental sustainability may be best obtained through what he calls a *post-growth, sustainable caring economy*.

 Hence, given the importance of care in formulating alternatives, I wish to complement Labonté’s editorial by elaborating on ‘care economies’ and the work of social reproduction. I begin by noting that the existing care economy is a structural part of capitalism that largely generates and sustains inequities. The ways in which capital organizes production and reproduction combine with systems of oppression by gender and race to generate vulnerability among the diverse populations.^3^ This reinforces Labonté’s argument that transformation is needed; that minor changes compatible with the growth imperative are unable to address the problems it creates. I then clarify the meaning of transformation in relation to care economies and the labor they entail. Transformation could, and should, change the perceived value, status, and material rewards of work in the care economy. It should shift the gender division of labor and reduce related socioeconomic inequality and gender inequality/inequities. Finally, I touch on the policy tools Labonté describes, highlighting how they connect to my broader point: that the care economy is currently an integral, but devalued part of capitalism; for a transformation to take place, raising perceived value, status, and relative material rewards of caring work and the people who do it must be an explicit policy goal.

## Care and transformation

 Care economies appear in Labonté’s comments in two forms: (*a*) as an economy that already exists and (*b*) as something to transition to — a future to be achieved. I’ll call the former the *care economy* and the latter a *Health and Social Care Economy* (HSCE). Linking the two is the transformation away from the existing political economy in which the capitalist pursuit of profits dominates the pursuit of human well-being.

 At present, profit-seeking drives production and consumption; it is the engine of capitalist growth. Growth has multiple sources but cost minimization, particularly minimizing the cost of the unique input — labor — is key. Profit-seeking incentivizes and exploits discrimination at multiple scales and reinforces inequities to reduce costs, for example by clustering marginalized populations into a smaller set of gendered and racialized jobs. Occupational segregation depresses wages and workers’ bargaining power in those jobs while reducing competition for higher status, better paid work. A care economy is present but is seen as marginal to ‘The Economy’; popularly imagined as production and paid work outside of the household. Care economy work is essential; however it is often low status, poorly paid or unpaid, and is disproportionately done by women. The care economy is therefore integral to socioeconomic inequality and inequities in the capitalist political economy.

 In a transformed world a different engine would displace the profit motive. The sustainable reproduction of life is a powerful alternative. It is already present in many of the activities undertaken by individuals in the care economy, such as childbirth (labor), childcare, eldercare and the day-to-day tasks typically done in the household. It is life-making. The transformation to a HSCE therefore hinges on changes to the perceived value, status, and material rewards of caring work — the work of *social reproduction*. A broadscale economy guided by the sustainable reproduction of life could offer a far more egalitarian economic system. Work would no longer need to be organized around profit maximization; its pay and status could reflect its *social value*. Where the profit motive incentivizes using inequities to enhance economic growth, its replacement could incentivize equity-enhancing production and reproduction of public goods. The incentive to deploy social oppressions to minimize costs by systematically paying, ie, women less than men would evaporate, at least in theory. Health, education, and social services, all sectors in which women are concentrated, could become the most highly valued and well-paid sectors of the economy, over the financial sector, for example. This possible HSCE should shift the gender division of labor and reduce related socioeconomic inequality and gender inequality/inequities.

 Such a transformation may seem implausible. The entrenched interests of capital have used inequities to serve capital accumulation, hence a transformation is likely to be resisted by entities that profit, or otherwise benefit, from inequities. Labonté notes that the immediate challenge to transformation is the rise of authoritarianism and the decline of democratic accountability. Authoritarianism, and conservatism more generally, are heavily invested in maintaining inequitable social relations. The maintenance of inequity is their raison d’être.^4^ The profit motive is compatible with authoritarianism; both rely on and reinforce inequities.

 However, a transformation *of some kind* seems inevitable. In the context of mass consumption primarily in the Global North and ecological devastation, the reproduction of life itself is increasingly unsustainable. Labonté notes several policy tools that could facilitate a socially desirable transformation. Women, the care economy, and the gender division of labor are missing from the policy discussion but are salient to conversations about tax justice, fiscal and monetary policy, and the lending practices of international financial institutions. I will return to this point after I elaborate on the substance of the existing care economy.

## Capitalism and the Existing Care Economy

 The care economy consists of the day-to-day work required to “maintain existing life and to reproduce the next generation.”^5^ Women are disproportionately tasked with this work through the gender division of labor. People are produced, both physiologically through women’s [going into] labor and through ongoing effortful activity done primarily by women. In this way, societies rely on women and their labor for their ongoing existence. Despite its obvious importance most care work is unpaid or poorly paid and relatively low status.

 Gender is central to the capitalist organization of work. It influences the paid and unpaid work activities that women and men are expected to take on. In unpaid work, the burden of reproductive labor on women increased during COVID-19, as is reflected in data about who left the labor force.^3^ During the pandemic, people, especially women, were forced to act as “shock absorbers” by providing home-based care for the sick and taking on additional household labor. However, pandemic damage mitigation expands the already-fraught work of reproducing life in non-pandemic conditions, potentially to the detriment of health generally and to women’s health in particular.^6,7^ It also increases women’s risk of exposure and reinfection at home.

 The care economy includes paid work in health, education, and social services. Women are concentrated in these sectors (ie, 85% of nurses and midwives are women globally) which also entail high risk of exposure.^9^ Higher infection rates for working-age (20-59 years) women are documented during COVID-19 peaks.^8^ In one case women were 80% of care workers but up to 90 percent of care workers with COVID-19. Just as societies rely fundamentally on women and their labor, healthcare systems depend fundamentally on women’s continued participation as suppliers of care.

 There is a substantial gender pay differential — globally, women earn 24% less than men — in the care sector, even after accounting for age, education, occupational category, working time, and public/private sector employment.^9^ Occupational demands, such as inflexible work schedules or long shifts, may conflict with women’s responsibility for care and other household work. Likewise, gendered responsibility for household work can limit women workers’ ability to meet occupational demands because paid occupations are not designed to accommodate realities of women’s lives.^6,7^ Many women in the paid care economy experience related distress and burnout. The household can be a dangerous worksite as well.^10^ Responsibility for care can be detrimental to one’s own well-being.^6,7^

 The paid and unpaid work in the care economy is therefore an integral part of socioeconomic inequality: the capitalist organization of work generates and reinforces inequities with material consequences. Therefore, in its present form, the massification of the care economy is not particularly appealing. However, the lamentable problems of reproductive labor and paid care work are emphatically *not* their existence. *The problems are the inequitable gender roles that task women with the work and the gendered value systems that leave it un- or poorly-compensated and devalued*.

## Policy for a Health and Social Care Economy

 The points above clarify that gender inequality/inequities are not “women’s issues.” They are social problems, constraints on the supply of care, and sources of systemic instability in healthcare and society. The pursuit of gender equity is crucial to any transition away from the existing organization of work. Without it, a transition is unlikely because of the low status of care as “women’s work” — but even if there were a transition, there is little reason to expect that the gender division of labor would change automatically or appreciably. In other words, for a transformation to take place, raising the perceived value of women and the work they are tasked with must be a policy goal. Policy, economic and otherwise, is not likely to change perceived value or the gender division of labor unless that change is an explicit aim for policy-makers.

 Labonté describes the disproportionate and negative impacts of COVID-19 on women as a rationale for greater public investment in health and social protection. He recognizes the consequences of the pandemic and of government policy on women that manifest through the gender division of labor. Yet women are missing from the discussion of policy — specifically taxes, modern monetary theory, and International Monetary Fund reform. Seemingly ‘gender-neutral’ policy is common, but policy rarely has gender-neutral outcomes. Gender analysis is needed to understand these effects.

 Despite the silence, progressive policy reform is typically directed at improving the conditions of social reproduction. For example, to Labonté the aim of global tax justice is the reallocation of accumulated wealth toward health and social benefits that could support people and households. At the macroeconomic level, low interest loans without the structural adjustment policies that are well-documented as exacerbating gender inequality/inequities could enable social spending in underdeveloped countries. At a micro/sectoral level, higher pay or additional benefits for care workers would contribute to socioeconomic equality and gender equality. They could also raise the status and perceived value of the work, making it more attractive to workers. Changing the perceived value, status, and material rewards of caring work and the people who do it is what would make the transformation transformative in people’s day-to-day lives.

 Finally, I have not touched on the question of degrowth policy/language or on reduced consumption of material goods in the Global North.^11^ The myriad other forms of sustainable, humanizing growth in capabilities and interdependency could be framed as regrowth. Higher ‘consumption’ of tangible and intangible things that improve quality life — like better health, time for leisure, solidarity, friendship — are likely to appeal to many people. Denaturalizing capitalism as the sole option in the popular imagination (of Global Northerners) can be empowering. I hesitate to use economistic language, but it could provide a bridge for a social rethinking what and who an economy, any economy, is *for*. Every society has an economy to provision life: the form and social possibilities beyond the profit motive are endless. There is much to be gained from valuing caring work and the people who do it.

## Ethical issues

 Not applicable.

## Competing interests

 Author declares that she has no competing interests.
